# Low Dose Carbon Black Nanoparticle Exposure Does Not Aggravate Allergic Airway Inflammation in Mice Irrespective of the Presence of Surface Polycyclic Aromatic Hydrocarbons

**DOI:** 10.3390/nano8040213

**Published:** 2018-03-31

**Authors:** Karina Lindner, Sina Webering, Michael Stroebele, Henning Bockhorn, Tanja Hansen, Peter König, Heinz Fehrenbach

**Affiliations:** 1 Institut für Anatomie, Zentrum für medizinische Struktur- und Zellbiologie, Universität zu Lübeck (UzL), Airway Research Center North (ARCN), Member of the German Center for Lung Research (DZL), 23562 Lübeck, Germany; lindner@anat.uni-luebeck.de (K.L.); koenig@anat.uni-luebeck.de (P.K.); 2 Forschungszentrum Borstel, Leibniz Lungen-Zentrum, Experimentelle Pneumologie, Airway Research Center North (ARCN), Member of the German Center for Lung Research (DZL), 23845 Borstel, Germany; swebering@fz-borstel.de; 3 Karlsruher Institut für Technologie, Engler-Bunte-Institut, Bereich Verbrennungstechnik, 76313 Karlsruhe, Germany; michael.stroebele@kit.edu (M.S.); henning.bockhorn@kit.edu (H.B.); 4 Fraunhofer Institut für Toxikologie und Experimentelle Medizin ITEM, Hannover, Biomedical Research in Endstage and Obstructive Lung Disease Hannover (BREATH), Member of the German Center for Lung Research (DZL), 30625 Hannover, Germany; tanja.hansen@item.fraunhofer.de

**Keywords:** asthma, allergic airway inflammation, mucociliary clearance, carbon black nanoparticles, polycyclic aromatic hydrocarbons, exacerbation

## Abstract

Exposure to exogenous noxae, such as particulate matter, can trigger acute aggravations of allergic asthma—a chronic inflammatory airway disease. We tested whether Carbon Black nanoparticles (CBNP) with or without surface polycyclic aromatic hydrocarbons (PAH) aggravate an established allergic airway inflammation in mice. In an ovalbumin mouse model, Printex^®^90 (P90), P90 coated with benzo[a]pyrene (P90-BaP) or 9-nitroanthracene (P90-9NA), or acetylene soot exhibiting a mixture of surface PAH (AS-PAH) was administered twice (70 µL, 100 µg/mL) during an established allergic airway inflammation. We analyzed the immune cell numbers and chemokine/cytokine profiles in bronchoalveolar lavages, the mRNA expressions of markers for PAH metabolism (Cyp1a1, 1b1), oxidative stress (HO-1, Gr, Gpx-3), inflammation (KC, Mcp-1, IL-6, IL-13, IL-17a), mucin synthesis (Muc5ac, Muc5b), the histology of mucus-producing goblet cells, ciliary beat frequency (CBF), and the particle transport speed. CBNP had a comparable primary particle size, hydrodynamic diameter, and ζ-potential, but differed in the specific surface area (P90 > P90-BaP = P90-9NA = AS-PAH) and surface chemistry. None of the CBNP tested increased any parameter related to inflammation. The unmodified P90, however, decreased the tracheal CBF, decreased the Muc5b in intrapulmonary airways, but increased the tracheal Muc5ac. Our results demonstrated that irrespective of the surface PAH, a low dose of CBNP does not acutely aggravate an established allergic airway inflammation in mice.

## 1. Introduction

Asthma is a common airway disease with more than 241 million prevalent cases worldwide in 2013 [1]. During an acute asthmatic response, the sufferer’s breathing is limited by an obstruction of the airway induced by smooth muscle constriction and mucus plugs in the airway lumen [2,3,4]. Moreover, asthmatic responses are associated with increased mucus secretion from goblet cells, epithelial injury, cellular infiltrations of immune cells, hyperplasia of smooth muscles, and deposition of excess collagen [2,5,6,7,8]. Asthma patients experience 0.3–3 acute aggravations of their symptoms per year—called asthma exacerbations—requiring an increase in their medication [9]. Because an exacerbation may require an emergency department visit or the hospitalization of the patient, asthma exacerbations are the main cost drivers of this chronic airway disease. Asthma exacerbations can be triggered by a variety of factors including air pollution [10].

Clinical and epidemiologic studies indicated that fine particles (<2.5 µm) and nanoparticles (<100 nm) from urban airborne particulate matter (PM) contributed to asthma exacerbation [11,12,13], showing an increased use of asthma medication [14], the reduction of lung function, and the increase of bronchial hyper-reactivity, as well as sputum IL-6 levels in subjects with asthma [13,15], after exposure to traffic-related air pollution.

The main sources of urban pollution are exhaust particles that contain carbonaceous particles with surface-bound substances such as polycyclic aromatic hydrocarbons (PAH) [16,17]. PAH are typically detected as complex mixtures containing low and high molecular weight PAH [18,19]. Low molecular weight PAH which have a 2- to 4-ring system—such as anthracene—are considered to be low-carcinogenic, whereas high molecular weight PAH—such as benzo[a]pyrene (BaP)—consist of more than 4 rings and are classified as high-carcinogenic [20]. The acute effects of PAH alone or when PAH are bound to Carbon Black nanoparticles (CBNP) comprise the induction of oxidative stress, cell death, and inflammation [21,22,23], indicating a possible contribution to asthmatic exacerbation [24].

Previous studies in animals, in which model allergens such as ovalbumin (OVA) were used to induce an allergic airway inflammation, showed that defined CBNP-such as Printex^®^90 (P90)-can aggravate the allergic airway inflammation [25,26,27] and hyperresponsiveness [28], and can increase the number of goblet cells in the intrapulmonary airways [25,26,28]. The CBNP used represent the carbonaceous core of exhaust particles [16,17] but do not reflect the physicochemical characteristics of naturally occurring particles that exhibit chemical surface modifications.

In the present study, we analyzed the acute effects of the defined CBNP to clarify whether surface-bound PAH enhance the potential of CBNP to trigger the aggravation of an established allergic airway inflammation and/or whether it may affect the mucociliary clearance. We used P90 as reference particles and coated them with PAH, either BaP or 9-nitroanthracene (9NA). Additionally, we tested soot that contained a mixture of surface-bound PAH generated from acetylene by flame synthesis [21].

Based on current reports that analyzed the content of fine and ultrafine particles in the air of European and Asian cities [29,30,31], and an experimental study that tested a low dose of P90 in a mouse model [32], we calculated a low dose exposure regimen for our experimental setting in order to avoid particle overload, which might overlap with any surface-specific particle effects. The CBNP were administered to OVA-sensitized animals during an acute asthmatic response induced by pulmonary OVA challenges. In contrast to previous studies [25,27], the asthma model used in our experiments reflects the three main features of asthma: airway hyperresponsiveness to methacholine, eosinophilic airway inflammation, and goblet cell metaplasia, which are aggravated by an additional trigger such as poly(I:C) [33]. Therefore, our experimental model might be superior for the assessment of the effects of CBNP in an asthmatic lung. Since airway remodeling associated with asthma differs in the proximal and distal airways, we not only analyze whether the CBNP aggravate the allergic airway inflammation due to their specific surface characteristics but also whether the reaction to the CBNP is different in the intrapulmonary and proximal airways.

## 2. Results

### 2.1. Characterization of CBNP and Their Suspensions

The CBNP were characterized with regards to the primary particle size, the specific surface area, the mass loss during heating up to 1000 °C, and their characteristics in aqueous suspension such as their hydrodynamic diameter and zeta (ζ)-potential. The data are presented in Table 1. The P90 nanoparticles and P90-9NA were already used in a previous study showing the transmission and scanning electron microscopic images and the particle size distribution of P90 particles, and the mass loss of P90-9NA as a function of the increased temperature [21]. In the present study, a new batch of P90-BaP and AS-PAH was used. Electron microscopic images and the size distribution of AS-PAH without the PAH, the mass loss up to 700 °C of P90-BaP and AS-PAH, and the PAH species identified on the surface of AS-PAH are shown in Figure S1 and Table S1.

All particles have a similar mean primary particle size, hydrodynamic diameter, and ζ-potential in water with 0.5% BSA. P90 exhibits a larger surface area and a smaller mass loss compared to the BaP- and 9NA-coated P90 particles and AS-PAH. The mass loss of P90-BaP and P90-9NA correlates with the content of BaP and 9NA adsorbed on the P90 surface. The identified PAH species that cover the surface of AS-PAH are shown in Table S1. We detected a mixture of at least 12 different PAH species with 2 to 5 aromatic rings. In the CBNP suspensions that were used, no endotoxins were measurable.

### 2.2. CBNP Did Not Increase Inflammatory Parameters in the Lungs of OVA-Treated Mice

**Figure 1:**
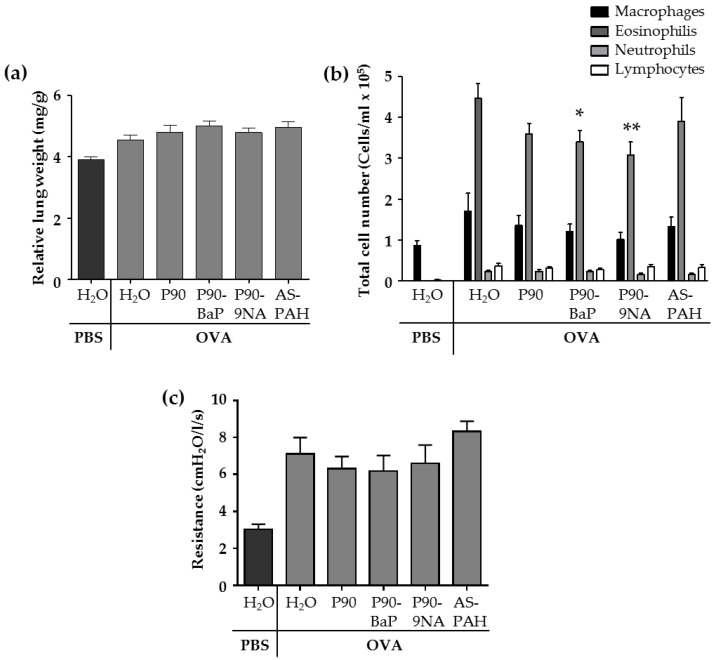
CBNP did not aggravate the inflammation in the lungs. The diagram in (**a**) shows the relative lung weight calculated from the quotient of the absolute wet weight of the left lung and the body weight of mouse (*n* = 13–18). The diagram in (**b**) shows the results of the differential cell count of immune cells detected in the brochoalveolar lavage (BAL) fluid (*n* = 18–25). The diagram in (**c**) shows the results of the airway resistance in response to 100 mg/mL methacholine (*n* = 7–8). All data are presented as mean ± SEM. The OVA/CBNP groups were compared to the OVA/H_2_O group and analyzed by the Mann Whitney U test. Significant changes were marked with * for *p* < 0.05 and ** for *p* < 0.01.

The allergic airway inflammation is associated with several changes in the lungs of animals of the OVA control group. Compared to the PBS control group, the relative lung weight (Figure 1a and Figure S2) and the parameters analyzed in the fluid from bronchoalveolar lavages (BALs)—the cell numbers of macrophages, lymphocytes, and granulocytes (Figure 1b), as well as the inflammation-related cytokines such as IL-5, IL-13, IL-6, IL-1β, KC, and Mcp-1 (Table 2)—were increased in the OVA control group. In addition, the airway hyperresponsiveness to methacholine was increased in the OVA control group compared to the PBS control group (Figure 1c).

The exposure to CBNP did not increase the relative lung wet weight (Figure 1a) nor the number of immune cells (Figure 1b) or inflammatory cytokines (Table 2) in the BAL fluid, and the airway hyperresponsiveness to methacholine was not different from OVA/H_2_O treated mice (Figure 1c).

However, we observed some significant decreases after CBNP-exposure compared to the OVA/H_2_O group: (i) P90 reduced IL-13 (Table 2); (ii) P90-BaP and P90-9NA decreased the number of eosinophils (Figure 1b), and reduced IL-4 (P90-BaP) or IL-1β and IL-13 (P90-9NA) (Table 2); (iii) AS-PAH reduced IL-4 and IL-1β (Table 2) in the BAL fluid. The reduction of Mcp-1 protein level in the BAL fluid was observed after exposure to all CBNP tested (Table 2).

### 2.3. CBNP Did Not Increase the mRNA Expression of Inflammatory-Relevant Markers in the Tracheal Epithelial Cells and Intrapulmonary Airways of OVA-Treated Mice

Since the airways and airway epithelial cells play a pivotal role in the modulation of the inflammatory response—for example, as a site of host defense and cytokine production—we analyzed the mRNA expression of markers for PAH metabolism, oxidative stress, and inflammation in tracheal epithelial cells and intrapulmonary airways.

PAH are metabolized by cytochrome P450 enzymes Cyp1a1 and Cyp1b1, resulting in toxic modifications. Our results demonstrated that the exposure to CBNP did not increase the mRNA expression of both Cyp enzymes in tracheal epithelial and intrapulmonary airways compared to the OVA/H_2_O group (Table 3).

A main effect of the nanoparticles is the induction of oxidative stress. Therefore, we analyzed the mRNA expression of oxidative stress markers glutathione peroxidase 3 (Gpx3), glutathione reductase (Gr), and heme oxygenase (HO-1). 

After CBNP-exposure, the mRNA expression of the three oxidative stress markers did not increase, compared to the OVA controls, neither in tracheal epithelial cells nor in intrapulmonary airways (Table 3).

The cells in the airways also contributed to the recruitment of immune cells into the lung by the release of inflammatory cytokines and chemokines. For this reason, we analyzed the mRNA expression of monocyte chemoattractant protein-1 (Mcp-1), an murine homolog of human IL-8 (KC), and the interleukins IL-6, IL-13, and IL-17a.

Our results showed that no CBNP increased the mRNA expression of the analyzed inflammatory markers (Table 3). Indeed, P90-9NA significantly decreased the mRNA expression of IL-6 and IL-13 in intrapulmonary airways compared to the OVA/H_2_O group (Table 3).

### 2.4. CBNP Did Not Increase the Number of Airway Epithelial Goblet Cells in OVA-Treated Mice

In asthma, the composition of the airway epithelium differs from healthy individuals showing, for example, an increased number of goblet cells filled with mucus. To determine the contribution of CBNP to mucus production, we analyzed the mRNA expression of the mucins Muc5b and Muc5ac, and the surface area covered by goblet cells and the volume of stored mucus in the intrapulmonary airways as well as the number of goblet cells in the tracheal epithelium using staining with periodic acid-Schiff (PAS) reagent.

Our results showed that the exposure to the PAH-containing CBNP, P90-BaP, P90-9NA, and AS-PAH, did not change the mRNA expression of the mucins compared to the OVA/H_2_O group (Table 3). However, P90 had different effects in the tracheal and intrapulmonary epithelial cells. P90 increased the mRNA expression of Muc5ac in the tracheal epithelial cells and decreased the mRNA expression of Muc5b in the intrapulmonary airways (Table 3).

**Figure 2:**
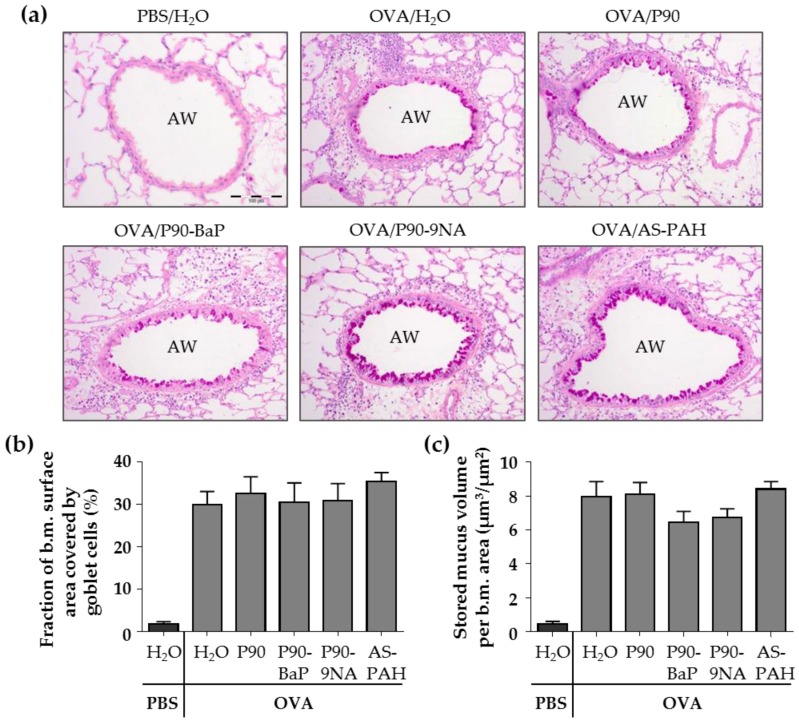

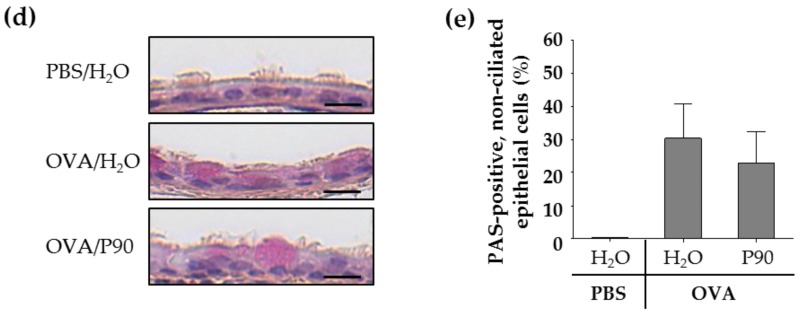
CBNP did not increase the number of PAS-positive cells in the airway epithelium. The images in (**a**) show the PAS-stained lung slices. PAS-positive cells are intensive pink and indicate mucus. AW means airway. Diagrams in (**b**) and (**c**) show the results of quantification, the fraction of the epithelial basement membrane (b.m.) surface area covered by goblet cells (**b**) and the stored mucus volume per b.m. area (**c**) (*n* = 7–8). The images in (**d**) show the PAS-stained tracheal epithelial cells. PAS-positive cells are pink and indicate mucus. The cell nuclei are blue (stained with hematoxylin). The quantification of PAS-positive non-ciliated cells is shown in (**e**) (*n* = 2–3).

The PAS staining of the lung slices showed that the exposure to CBNP did not increase the surface covered by goblet cells nor the stored mucus volume in intrapulmonary airways compared to the OVA/H_2_O group (Figure 2a–c). Since P90 increased the Muc5ac mRNA expression in the tracheal epithelial cells, we additionally stained the tracheal epithelium after exposure to P90 with PAS. The results showed that the number of PAS-positive cells was not increased after P90-exposure compared to the OVA/H_2_O group (Figure 2d,e).

### 2.5. P90-Induced Mucus Release Affects the Ciliary Beat Frequency in Tracheal Epithelial Cells of OVA-Treated Mice

**Figure 3:**
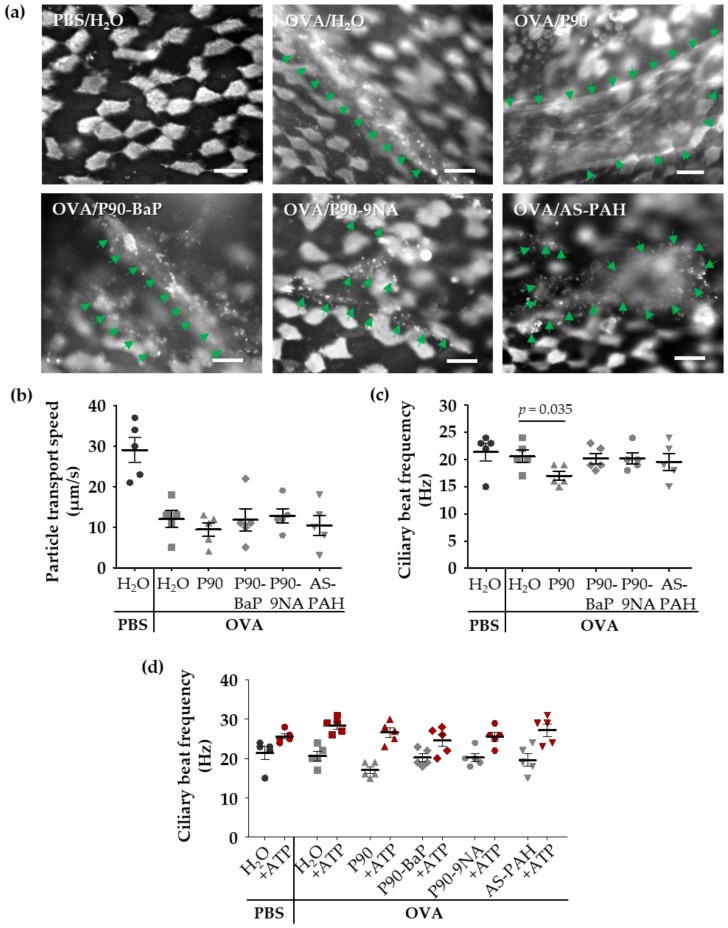
P90 aggravates the effects on mucociliary clearance in the trachea. The images in (**a**) show the mucus and ciliated cells (both are white) after staining with fluorescent lectins: Ulex europaeus agglutinin-1 and wheat germ agglutinin. The green arrows indicate mucus structures (*n* = 3). Diagrams in (**b**–**d**) show the results of the analysis of particle transport speed (**b**) and the ciliary beat frequency (**c**,**d**) after exposure to CBNP. Every data point indicates a single experiment (**b**–**d**). The red symbols in (**d**) indicate the results of the stimulation with ATP. The mean ciliary beat frequency was calculated from at least 40 (**c**) and 30 (ATP stimulation in (**d**)) ciliated cells measured at four (**c**) or three (ATP stimulation in (**d**)) different tracheal regions of each animal. The lines with bars indicate the mean ± SEM.

To visualize the released mucus on the epithelium, we stained the mucus with fluorescent lectins in freshly explanted tracheae. The lectin staining showed increased mucus amounts on the epithelium of all OVA-treated mice (Figure 3a). However, the staining of mucus after exposure to P90 indicated an increased mucus quantity on the tracheal epithelium compared to the OVA controls (Figure 3a). Scanning electron microscopic images supported this finding, showing a mucus-covered epithelium after P90-exposure (Figure S3a).

Scanning electron microscopic images not only showed mucus on the tracheal epithelium after OVA treatment, but it also showed dead epithelial cells (Figure S3a). Since the mucus and the damaged epithelial cells can interfere with cilia-driven clearance in the airways, we analyzed the mean ciliary beat frequency and mean particle transport speed, and quantified the dead cells on the tracheal epithelium.

Compared to the PBS control group, the cilia-induced particle transport speed was decreased in the OVA controls (Figure 3b), whereas the ciliary beat frequency was not affected (Figure 3c), indicating that the mucus on the epithelium decelerated the transport speed. The exposure to the CBNP did not further decrease the particle transport (Figure 3b), but the ciliary beat frequency was decreased following exposure to P90 (Figure 3c). 

To clarify whether this effect was due to a P90-triggered damage to the epithelial cells, we analyzed the dead epithelial cells in whole mount preparations of the trachea (Figure S3b). Generally, we detected no apoptotic cells in the whole mount preparations. However, the amount of necrotic epithelial cells was increased in the OVA control group compared to the PBS controls (Figure S3c). The exposure to CBNP, however, did not lead to a further increase in the number of necrotic epithelial cells (Figure S3c).

Due to these findings, we added adenosine triphosphate (ATP) to the trachea samples to assess the functionality of the cilia. After ATP-exposure, the ciliary beat frequency was equally increased in all test groups (Figure 3d) indicating that P90 did not induce a cilia damage.

Since the ciliary beat frequency directly correlates with the particle transport speed without mucus or dead cells on the epithelium, the particle transport speed was only increased in the PBS controls after ATP-exposure (Figure S4). In the trachea of OVA-treated mice (with or without exposure to CBNP), the increased ciliary beat frequency did not result in an increase of the particle transport speed (Figure S4), most likely due to the extensive amounts of mucus on the epithelium.

## 3. Discussion

In a previous study, we showed that CBNP coated with PAH had only minor pro-inflammatory effects in healthy animals in vivo [21]. However, CBNP might exert different responses in individuals suffering from lung diseases, such as allergic asthma, and might result in the induction of an acute asthma exacerbation. In the present study, we showed that P90, with or without a defined surface-PAH and an acetylene soot with a mixture of surface PAH, did not acutely aggravate the established allergic airway inflammation in mice. However, the unmodified P90 particles increased the mRNA expression of Muc5ac in tracheal epithelial cells, which is in agreement with the notion that a more adhesive and viscous Muc5ac-containing mucus is released [34].

In this study, we used P90 nanoparticles and coated the surface with a high-mutagenic 5-ring PAH (that is, benzo[a]pyrene, BaP) or a low-mutagenic 3-ring PAH (that is, 9-nitroanthracene, 9NA). Since the particle characteristics, such as the particle size and surface properties, define the cytotoxic potential of particles [35], we analyzed the particle characteristics under dry conditions and in aqueous suspension. As also previously described in Reference [21], all P90-based nanoparticles have a similar mean primary particle size in the dry state and a similar hydrodynamic diameter and ζ-potential in water with 0.5% BSA. However, due to the coating procedure, the specific surface area of the coated P90 particles is only approximately one-third of the surface area of unmodified P90 particles. The content of PAH on the P90 surface was 10% BaP (P90-BaP) or 14% 9NA (P90-9NA) of the total particle mass. The similar PAH content and specific surface area of coated P90 particles allowed us to analyze the PAH-specific effects on the airway epithelial cells.

Additionally, we tested soot from acetylene combustion (AS-PAH) that reflects carbon-based nanoparticles from naturally occurring combustion processes. Compared to the coated P90 nanoparticles, the AS-PAH had similar particle characteristics regarding particle size, hydrodynamic diameter, and ζ-potential. However, AS-PAH contains a mixture of at least 12 PAH species on the surface with 2 to 5 aromatic rings. We identified the PAH acenaphthylene, which contains 3 aromatic rings, as the most abundant and the PAH cyclopenta[cd]pyrene, which consists of 5 aromatic rings, as the most toxic. Compared to the 5-ring PAH BaP, cyclopenta[cd]pyrene induces the same mutation mechanism [36] and is more tumorigenic in mouse studies [37]. However, based on the identified PAH species on the AS-PAH surface and our previous findings [21], the AS-PAH is potentially the most toxic nanoparticle tested in this study.

To avoid unrealistically high CBNP concentrations, we calculated a CBNP dose based on studies that determined the PM_2.5_ concentration in the ambient air of several cities in Europe and Asia [38,39,40,41,42,43,44]. In European cities, the mean annual concentration of PM_2.5_ fraction is low and ranged between 7 µg/m^3^ and 35 µg/m^3^ in the last few years [38,39,40,41,42]. However, the PM_2.5_ fraction can be temporally increased—for example, in congested roads—up to 76 µg/m^3^ [13]. In Chinese cities, the mean annual concentration of PM_2.5_ is higher and ranged from 55 µg/m^3^ to 102 µg/m^3^ in 2013 [43,44]. Moreover, the temporary peak concentrations can reach levels over 600 µg/m^3^ in urban areas [45,46].

For our calculation, we used the data given by the Mouse Phenome Database for female Balb/c mice [47], that is, a minute volume of 98 mL/min results in an inhaled amount of approximately 140 liters air/day. At a concentration of 50 µg/m^3^, a mouse inhales approximately 7 µg PM_2.5_/day. Regarding the deposition efficiency of inhaled ultrafine particles in Balb/c mice and human lungs, only 40–50% of inhaled particles (in our case, 2.8 µg–3.5 µg PM_2.5_) were deposited in the lungs [48,49].

At the dose of 7 µg used in this study, the CBNP did not induce an acute increase of inflammation in the lungs or related parameters in the intrapulmonary airways or tracheal epithelial cells. This result is in agreement with the findings of a clinical double-blinded randomized cross-over pilot study that also found no acute effects on the allergic inflammation in human lungs after exposure to CBNP [50]. However, the authors suggested a long-term effect of CBNP on the course of the asthmatic disease, as described for P90 nanoparticles in an allergen (OVA) asthma mouse model [25]. The results obtained from the mouse model showed that the time point of P90-exposure determines the acute effect on inflammation and the course of the inflammatory response. If mice received the P90 nanoparticles 24 h before the allergen challenge and thus, before the establishment of an allergic airway inflammation, the acute inflammatory response to allergen-exposure was pronounced [25]. In contrast, if mice received the P90 nanoparticles 24 h after the establishment of an allergic airway inflammation, the inflammatory infiltrates were initially decreased and exhibited a temporally delayed increase after seven days [25].

In our study, we did not detect an increase or a decrease in immune cell numbers after exposure to P90 nanoparticles. Since we used a more distinctive asthma model with pronounced eosinophilia that indicated a present inflammation, and repeated CBNP-exposure, immediately before and 24 h after the last OVA challenge. Our more complex stimulation strategy may contribute to these differences.

Because we previously demonstrate that the response of lungs and tissues of healthy animals depends on the specific surface chemistry (that is, PAH) of the CBNP [21], we tested if the same holds true for animals suffering from an inflammatory lung disease such as asthma. We found that in contrast to P90, P90-BaP and P90-9NA decreased the numbers of eosinophils in the lungs, indicating that the coating might contribute to the previously described long-term effects on the course of allergic asthma diseases [25]. 

Immune and epithelial cells of the lung can respond to inhaled substances by the release of central mediators of asthma, such as IL-4 and IL-13 [51], as well as inflammatory mediators, such as IL-1β [52], and immune cell attracting proteins, such as Mcp-1 [53]. The profile of the mediators released depends on the inhaled substance and can be detected in the BAL fluid. In our study, the profile of inflammatory mediators was differentially regulated by the CBNP. The P90 nanoparticles decreased the protein level of IL-13, P90-BaP reduced the protein level of IL-4, P90-9NA induced the decrease of IL-1β and IL-13, and AS-PAH decreased the protein levels of IL-4 and IL-1β. Since AS-PAH contained a PAH-mixture with 3-ring and 5-ring PAH and decreased IL-4 like P90-BaP—which is coated with the 5-ring PAH BaP—and IL-1β like P90-9NA—which is coated with the 3-ring PAH 9NA—we suggest that the modulation of inflammation depends on the chemistry of the surface PAH. However, the decreased levels of the Mcp-1 protein after exposure to all CBNP, including the uncoated P90 particles, suggest that the general particle characteristics such as the particle size or the ζ–potential contribute to the modulatory effect. In summary, our data indicate that the tested CBNP attenuate the allergic airway inflammation in mice. Although this response seems to be beneficial at first sight, it must be taken into account that this modulation of the immune response may affect the following reactions to allergens, leading to the worsening of asthmatic diseases [25]. 

Previous studies indicated that a lowered antioxidant defense may be responsible for the increased susceptibility to CBNP and CBNP-induced acute exacerbations of allergic airway inflammation [24,54]. However, our results did not confirm these reports. Although the antioxidant enzyme Gpx-3 had a decreased mRNA level after the OVA challenge, neither P90 nor the PAH-containing CBNP (P90-BaP, P90-9NA, or AS-PAH) induced the mRNA expression of cytokines or chemokines in the tracheal epithelial cells. Moreover, the CBNP also did not exacerbate the inflammatory response in the intrapulmonary airways. Only P90-9NA showed modulatory effects, decreasing the mRNA expression of the inflammatory mediator IL-6 and, consistent with the decreased IL-13 protein levels in the BAL fluid, of IL-13.

Besides airway inflammation, the increase of mucus secretion from goblet cells is a main feature of the acute asthmatic response. Indeed, mucus plugging was suggested to be a major factor responsible for the reduced lung function in severe asthmatics [55]. Mucus can be stained in the goblet cells with periodic acid-Schiff (PAS) reagent. The periodic acid oxidizes the carbohydrates on mucins, mainly Muc5ac, producing aldehyde groups that condense with the Schiff’s reagent and result in a magenta coloration. Several studies showed that CBNP can increase the number of PAS-positive goblet cells in the airway epithelium in allergen (OVA) treated mice [25,27,28,56], indicating an increased amount and release of mucus. In our present study, we did not find increased numbers of PAS-positive goblet cells or an increased volume of stored mucus in the airway epithelium after exposure to the CBNP. 

However, the main gel-forming mucins in mammals are Muc5ac and Muc5b [34]. Muc5b has physiologic functions, ensuring normal mucus clearance, whereas Muc5ac plays a role in mechanisms of allergen-induced airway hyperresponsiveness, mucous metaplasia, and airway mucus plugging [57,58,59]. After exposure to unmodified P90 nanoparticles, we detected an increase of the Muc5ac at the mRNA level in the tracheal epithelial cells compared to the OVA/H_2_O group. In asthma, it is known that Muc5ac-rich mucus impairs the cilia-driven transport by epithelial tethering without defects in the cilia function [60]. Based on our results, we suggest that the further increase of Muc5ac induced by P90 nanoparticles modified the composition and characteristics of the released mucus. The resulting more adhesive and viscous mucus might have agglutinated the cilia, causing the observed decrease in the ciliary beat frequency. Since the PAH-coated P90 particles and the AS-PAH did not increase the mRNA expression of Muc5ac and did not affect the ciliary beat frequency, the higher specific surface area of P90 nanoparticles might be the critical particle characteristic responsible for this epithelial response. 

Our results additionally revealed an airway-region specific response to P90. While the Muc5ac mRNA expression was increased in the tracheal epithelial cells, the Muc5ac mRNA levels in the intrapulmonary airways remained constant. In contrast, P90 decreased the mRNA expression of Muc5b in the intrapulmonary airways but had no effect on Muc5b mRNA level in the tracheal epithelial cells. As mentioned above, Muc5b, besides Muc5ac, is the main mucus-forming protein in the airways. Muc5b is required for the airway defense against pathogens and is important for effective mucociliary clearance [57,61]. Our results indicate that the proportion of Muc5ac and Muc5b is affected due to the P90-induced decrease of Muc5b mRNA expression in the intrapulmonary airways, probably affecting the mucociliary clearance. Since the decrease of the Muc5b expression is predicted to create an environment that promotes eosinophil survival [59] and, furthermore, the reduced Muc5b expression is a feature of severe (Th2-high) asthma [62], the P90-induced reduction of Muc5b mRNA levels also potentially indicates the worsening of allergic airway inflammation. 

Since we administered the CBNP during an acute asthmatic response with extensive mucus release, the hydrophilic and lipophilic features of the CBNP may play a role in bioavailability [63]. The hydrophilic property of P90 may help the particles to penetrate into the mucus layer and facilitate the contact with the airway epithelium. In contrast, the lipophilic features of PAH bound on the surface of P90 particles and AS-PAH could facilitate the contact to glycoproteins and lipids in the mucus, leading to reduced bioavailability [63]. 

In conclusion, our results demonstrate that a relatively low dose of CBNP with or without PAH on the surface did not acutely aggravate the allergic airway inflammation in a mouse model of experimental allergic asthma. However, the PAH coating changed the biologic effect of CBNP on immune reaction and airway epithelial cells in vivo.

## 4. Materials and Methods 

### 4.1. Particle Preparation and Characterization

Printex^®^90 (P90) was used as a reference CBNP. The surface was modified with benzo[a]pyrene (BaP) or 9-nitroanthracene (9NA). Additionally, we used soot obtained by acetylene combustion that contained a mixture of PAH on the surface. The coating procedure, the production of acetylene soot, and the methods of CBNP characterization are previously described in Reference [21]. 

### 4.2. Preparation and Characterization of CBNP Suspension 

The administered CBNP suspensions had a concentration of 100 µg/mL, prepared with a 10 mg CBNP sample and 100 mL of double-deionized water. For suspension stability, 500 mg of bovine serum albumin (BSA) (AppliChem Inc., Maryland Heights, MO, USA) was added. The suspensions were homogenized with an ultrasonic homogenizer as previously described in Reference [21]. The CBNP suspensions were used for experiments if no endotoxin was detectable using the CROMO-LAL assay (Associates of Cape Cod Inc., East Falmouth, MA, USA) following the manufacturer’s protocol. The particle size and ζ-potential were measured with a Malvern Zetasizer Nano-ZS (Malvern Instruments Ltd., Worcestershire, UK) at room temperature. The results are presented as mean ± SEM.

### 4.3. Animals

Female 7–9 weeks old Balb/c mice (Charles River Laboratories, Sulzfeld, Germany) were housed under standard conditions. The mice received an OVA-free diet and water ad libitum. The in vivo studies were approved by the Ministerium für Energiewende, Landwirtschaft, Umwelt und ländliche Räume des Landes Schleswig-Holstein (V242-7224.122-1 (13-1/15) and V244-7224.121.3 (12-1/15)).

### 4.4. Animal Treatment Protocol

The experimental allergic asthma model was previously described in Reference [64]. The mice were sensitized to ovalbumin (OVA; albumin from chicken egg white, Grade VI; Sigma-Aldrich Chemie GmbH, Munich, Germany) by three intraperitoneal (i.p.) injections of OVA adsorbed to aluminium hydroxide (Imject™ Alum Adjuvant, Thermo Scientific Inc., Pierce Biotechnology, Rockford, IL, USA) on day 1, 14, and 21. The control group received 200 µL Dulbecco’s phosphate-buffered saline i.p. (D-PBS; Sigma-Aldrich Chemie GmbH, Munich, Germany). On day 26, 27, and 28, the animals were exposed to an OVA (Grade V; Sigma-Aldrich Chemie GmbH, Munich, Germany) aerosol (1% *w*/*v* in PBS). The oropharyngeal application of CBNP suspension was performed on day 28 before the last challenge and on day 29. For this procedure, the mice were anesthetized with isoflurane and received 70 µL of CBNP dissolved in sterile water (100 µg CBNP/mL water) or only water as a control. On day 30, the mice were euthanized and the body and lung weight was determined.

### 4.5. Differential Cell Count in Bronchoalveolar Lavage (BAL) Fluid

Before explantation of airways or trachea, the trachea was cannulated and the lungs were rinsed with 1 mL D-PBS (Sigma-Aldrich Chemie GmbH, Munich, Germany) containing a protease inhibitor (Complete, Roche, Basel, Switzerland). The volume of BAL fluid was recorded and the number of immune cells was determined using a Neubauer counting chamber. For differential cell count, 50 µL of BAL fluid was cytospinned (500× *g*, 5 min; Cytospin™, Thermo Scientific GmbH, Schwerte, Germany) and the immune cells (macrophages, lymphocytes, eosinophil and neutrophil granulocytes) were stained using the Pappenheim method or the Diffquick solutions (Medion Grifols Diagnostics AG, Düdingen, Switzerland). A total number of at least 100 immune cells was counted. The results are presented as the total number of macrophages, eosinophils, neutrophils and lymphocytes per mL BAL fluid.

### 4.6. Preparation of Airways

The tracheae were explanted, transferred to culture dishes that was coated with a thin layer of Sylgard^®^184 Silicone Elastomer (Dow Corning GmbH, Wiesbaden, Germany), and filled with HEPES-buffered Ringer solution. The tracheae were fixed on the dish bottom with insect needles and prepared for the isolation of epithelial cells and, following RNA isolation, for mucus staining and functional experiments. The distal airways were microdissected from the surrounding parenchyma under a stereomicroscope and pooled for RNA experiments [65].

### 4.7. RNA Isolation and Quantitative RT-PCR

The isolation of the total RNA from isolated tracheal epithelial cells and dissected distal airways, the used reagents for DNA digestion and reverse transcription, and the PCR conditions are previously described in Reference [21]. 

For the determination of the mRNA expression levels in tracheal epithelial cells, the real-time RT-PCR was performed in TaqMan Universal PCR Master Mix (Life Technologies GmbH, Darmstadt, Germany) using the ABI PRISM 7900HT or StepOnePlus™ Sequence Detector System (both Applied Biosystems, Foster City, CA, USA). The probes were tagged with reporter 6-FAM and quencher Tamra. Changes of the mRNA expression were determined by calculating the difference in the cycle threshold to the housekeeping gene RPL32 with the ΔΔC_T_ method and represented as an n-fold expression (2^−ΔΔC^_T_).

To analyze the changes in the transcript levels in distal airways, the real-time RT-PCR was performed on a Roche Light Cycler 480 II Instrument (Roche Diagnostics Deutschland GmbH, Mannheim, Germany) using Light Cycler 480 SYBR green I Master (Roche Diagnostics Deutschland GmbH) according to the manufacturer’s protocol. The PCR cycling conditions were as follows: one cycle at 95 °C for 10 min followed by 45 cycles touch-down 63 °C–58 °C for 8 s with 0.5 °C/s and 72 °C for 10 s. Changes in mRNA expression were determined by calculating the n-fold difference in the cycle threshold to the housekeeping gene RPL32 by normalization using the rule of proportion.

The probes and primers used for quantitative real-hypertime RT-PCR are shown in Table S2.

### 4.8. Measurement of the Protein Levels in the BAL Fluid

After centrifugation of the BAL fluid, cytokines and chemokines relevant for lung inflammation were determined in the supernatant by using a Cytometric bead array (CBA Flex Set Kits; BD Bioscience, San Jose, CA, USA) according to the manufacturer’s protocol. The fluorescent intensity was analyzed on a BD Accuri™ C6 flow cytometer (BD Bioscience, San Jose, CA, USA). The analysis was performed using the FCAP Array™ v3.0 Software (BD Bioscience, San Jose, CA, USA). The results are presented as absolute protein concentrations (pg/mL).

### 4.9. PAS Staining

The lungs were fixed ex situ by the intra-tracheal instillation of a 4% (*w*/*v*) phosphate buffered paraformaldehyde. Afterwards, the lungs were removed, stored in 4% paraformaldehyde overnight, and embedded in paraffin. The orientation of the lungs was randomized according to the orientator technique [66]. In order to quantify the goblet cells and stored mucus in the airway epithelium, 2 µm thick lung sections were stained with periodic acid-Schiff (PAS) reagent. Images were recorded using a microscope (BX-51; Olympus, Tokyo, Japan) equipped with a digital camera (DP-25; Olympus, Tokyo, Japan) at 20-fold magnification. The surface area of the mucin-containing goblet cells (*S*_gc_) per total surface area of airway epithelial basal membrane (*S*_ep_) and the volume of the PAS-stained epithelial mucin (*V*_mucin_) per *S*_ep_ were determined using a computer-assisted stereology toolbox (newCAST, Visiopharm, Hoersholm, Denmark) that was previously described in Reference [67]. *S*_gc_, *S*_ep_, and *V*_mucin_ were calculated according to the formulas: $S_{gc}/S_{ep}=\sum I_{gc}/\sum I_{ep}$ and $V_{mucin}/S_{ep}=LP\times \sum P_{mucin}/2\times \sum I_{ep}$, where $I_{gc}$ is the interaction of the test line with the goblet cells, $I_{ep}$ is the interaction of the test line with the epithelial basal membrane, $P_{mucin}$ is the point hitting mucin, and LP is the test line length at final magnification. 

Trachea samples were explanted, pinned on a cork plate, and fixed in 4% phosphate-buffered paraformaldehyde. The trachea samples were embedded in paraffin and longitudinally sliced in fractionated series. The 7 µm thick sections were stained via PAS reaction and Hematoxylin. The PAS-positive cells were counted in a minimum of 2 different areas with a distance of 490 µm. The areas had a minimum width of 350 µm analyzing a slice every 70 µm. A minimum of 520 total epithelial cells was counted in the areas, respectively. The amount of PAS-positive cells is presented as the percentage of the total number of non-ciliated cells.

### 4.10. Mucus Staining on the Vital Tracheal Epithelium

The tracheae were incubated in 1 mL of HEPES-buffered Ringer solution at 30 °C. Next, 0.5 µL of wheat germ agglutinin (WGA) and Ulex europaeus agglutinin both labeled with Alexa Fluor^®^ 555 (both 1 µg/mL; Molecular Probes Inc., Eugene, OR, USA) were added. Both lectins stain carbohydrate residues present in mucins [68,69] and WGA also stains cilia [70]. The staining was imaged with a Zeiss Examiner.D1 microscope equipped with an AxioMR camera and an Achroplan 40×/0.8 W objective (Carl Zeiss MicroImaging GmbH, Göttingen, Germany).

### 4.11. Whole Mount Analysis of Dead Cells

The whole mount samples of the tracheae were incubated in a pre-warmed HEPES-buffer Ringer solution. Ethidium homodimer-1 (1 µg/mL; Life Technologies GmbH, Darmstadt, Germany) that stains necrotic cells was added and the trachea samples were incubated for 30 min at 37 °C. The samples were fixed with a 4% paraformaldehyde (pH 7.4), washed with PBS (pH 7.4), and permeabilized with ice-cold acetone. The samples were incubated with two antibodies overnight at room temperature, a rabbit monoclonal anti-cleaved caspase-3 antibody (Clone 5A1E, dilution 1:200, Cell Signaling Technology Inc., Danvers, MA, USA) that was labeled with Atto488 (Lightning-Link™Atto488 conjugation kit; Innova Bioscience Ltd., Cambridge, UK), and a mouse monoclonal anti-acetylated α-tubulin antibody (Clone 6-11B-1, dilution 1:200; Sigma-Aldrich GmbH, Steinheim, Germany) that was labeled with Atto647 (Lightning-Link™Atto647 conjugation kit; Innova Bioscience Ltd., Cambridge, UK). After washing with PBS (pH 7.4), the whole mount preparations were incubated with Hoechst 33258 (1 µg/mL; Sigma Aldrich, Steinheim, Germany) for 1 h at room temperature. Next, the trachea samples were washed again with PBS (pH 7.4) and covered with Mowiol mounting medium. The anti-cleaved caspase-3 antibody labels apoptotic cells and the anti-acetylated α-tubulin antibody labels cilia for the identification of the epithelial layer. Cell nuclei were stained with Hoechst 33258. Apoptotic and necrotic epithelial cells were localized using a confocal laser scanning microscope (LSM 710; Carl Zeiss MicroImaging GmbH, Göttingen, Germany) equipped with a Plan-Neofluor 40×/1.30 oil DIC objective. A minimum of 2700 epithelial cells was counted in the complete whole mount preparation. The results are presented as a percentage of the total cell numbers.

### 4.12. Particle Transport Speed and Ciliary Beat Frequency

Trachea samples were transferred to a Delta T4 Culture dish (Bioptechs Inc., Butler, PA, USA) whose bottom was covered with a Sylgard^®^184 Silicone Elastomer. The samples were fixed on the bottom with the epithelium facing upwards and covered with 2 mL of HEPES-Ringer solution. The incubation temperature was maintained at 30 °C using a Bioptechs Delta T4 Culture Dish Controller (Bioptechs Inc., Butler, PA, USA). The particle transport and the cilia beating were imaged using a Zeiss Examiner.D1 microscope equipped with an Achroplan 40×/0.80 W or 20×/1.0 DIC (UV) VIS-R objectives (Carl Zeiss MicroImaging GmbH, Göttingen, Germany). The transport of 4.5 µm polystyrene particles (2 × 10^6^ particles/mL) and the cilia motion were recorded with an SMX-150M camera (EHD Imaging GmbH, Damme, Germany).

Particle transport was recorded with 200 images at a frame rate of 12 Hz from at least four different areas for each trachea. The particle transport speed was determined using Image-Pro^®^Plus 6.0 (Medium Cybernetics, Inc., Bethesda, MD, USA) and the mean particle transport speed was calculated for each analyzed trachea. 

The cilia motion was recorded with 1000 images at a 100 Hz frame rate in at least four different areas for each trachea. The ciliary beat frequency of 10 ciliated cells was measured in every area. The ciliary beat frequency was determined by Fourier transformation of grey level changes over time using a Mathlab-based software (Mathworks, Natick, MA, USA) and the mean frequency was calculated for each trachea. To activate cilia beating, adenosine triphosphate (ATP, 10 µM) was added at the end of each experiment.

### 4.13. Assessment of Airway Responsiveness to Methacholine

The airway responsiveness to methacholine (MCh) was invasively assessed by measurement of the total lung resistance using Finepoint RC Units (Data Science International, New Brighton, MN, USA). The mice were anesthetized, tracheotomized with a cannula, and mechanically ventilated as previously described in Reference [33]. The data represent the airway resistance in response to a 100 mg/mL MCh dose.

### 4.14. Preparation of Trachea Samples for Scanning Electron Microscopy

After fixation in the Monti-Graziadei solution (pH 3.5) [71], the trachea samples were washed with 0.1 M sodium cacodylate buffer (pH 7.4) and subjected to critical-point drying. The dried samples were sputtered with platinum (Polaron SEM coating system, Polaron Instruments, Lewes, UK) and the tracheal epithelium was investigated using a scanning electron microscope (EVO HD15, Zeiss, Oberkochen, Germany).

### 4.15. Statistical Analysis

The data are presented as mean ± SEM. All experiments were carried out at least three times. The results were analyzed using the Mann-Whitney U test in GraphPad Prism (GraphPad Software, Inc., La Jolla, CA, USA). *p*-values < 0.05 were considered statistically significant.

## Figures and Tables

**Table 1:** Particle characteristics.

Particle Characteristics	Printex^®^90 (P90)	P90 Coated with benzo[a]pyrene (P90-BaP)	P90 Coated with 9-Nitroanthracene (P90-9NA)	Acetylene Soot with PAH-Mixture (AS-PAH)
Mean primary particle size (nm)	16.5 ± 0.4	16.5 ± 0.4 *	16.5 ± 0.4 *	14.4 ± 0.3 *
Specific surface area (m^2^/g)	302 ± 16	120 ± 2	91 ^#^	124 ± 2
Mass loss up to 1000 °C (%)	0.5 ± 0.4	10.0 ± 0.2	14.6 ± 0.1	15.3 ± 0.2
Hydrodynamic diameter in water/BSA (nm)	166 ± 9	163 ± 6	161 ± 3	163 ± 6
ζ-potential in water/BSA (mV)	−33 ± 1	−32 ± 1	−33 ± 1	−32 ± 1

Data are mean ± SEM. *n* = 3–6, except ^#^ *n* = 1, * measurement without surface modifications.

**Table 2:** Cytokine and chemokine levels in the bronchoalveolar lavage (BAL) fluid.

Protein	PBS/H_2_O	OVA/H_2_O	OVA/P90	OVA/P90-BaP	OVA/P90-9NA	OVA/AS-PAH
Mcp-1	0.01 ± 0.01	27.0 ± 5.7	12.7 ± 3.9 *	7.4 ± 5.5 *	11.7 ± 5.7 *	0.6 ± 0.3 *
KC	9.5 ± 2.3	21.6 ± 5.7	16.7 ± 4.3	17.7 ± 4.2	21.3 ± 7.9	9.9 ± 2.5
IL-1β	0.06 ± 0.02	1.3 ± 0.2	0.8 ± 0.2	0.8 ± 0.2	0.7 ± 0.2 *	0.5 ± 0.1 *
IL-6	0.4 ± 0.1	1.1 ± 0.3	0.8 ± 0.2	0.7 ± 0.1	0.7 ± 0.2	0.7 ± 0.2
IL-4	0.8 ± 0.6	1.2 ± 0.2	0.7 ± 0.1	0.7 ± 0.1 *	0.9 ± 0.2	0.6 ± 0.2 *
IL-5	0.8 ± 0.3	12.7 ± 2.1	10.4 ± 1.8	13.4 ± 1.8	11.1 ± 1.8	11.7 ± 2.2
IL-13	0.03 ± 0.01	7.3 ± 1.1	4.4 ± 0.9 *	5.7 ± 1.1	4.4 ± 0.6 *	5.4 ± 1.2
IL-17A	0.2 ± 0.1	0.4 ± 0.1	0.3 ± 0.1	0.3 ± 0.1	0.4 ± 0.1	0.3 ± 0.1

The concentration unit is pg/mL. Data are presented as mean ± SEM (*n* = 11–16). The OVA/CBNP groups were compared to the OVA/H_2_O group and analyzed with the Mann Whitney U test. Significant changes were marked with * for *p* < 0.05.

**Table 3:** The mRNA expression of the relevant markers for PAH metabolism, oxidative stress, and inflammation.

Gene	Airway Section	PBS/H_2_O	OVA/H_2_O	OVA/P90	OVA/P90-BaP	OVA/P90-9NA	OVA/AS-PAH
***Cyp1a1***	intrapulmonary airways	1.0 ± 0.1	1.0 ± 0.2	1.3 ± 0.4	1.3 ± 0.2	0.7 ± 0.1	1.5 ± 0.3
	tracheal epithelial cells	bld	bld	bld	bld	bld	bld
***Cyp1b1***	intrapulmonary airways	1.0 ± 0.1	0.6 ± 0.1	0.6 ± 0.1	0.8 ± 0.1	0.5 ± 0.1	0.9 ± 0.1
	tracheal epithelial cells	1.1 ± 0.2	1.2 ± 0.3	1.4 ± 0.3	1.3 ± 0.4	1.6 ± 0.7	2.8 ± 0.9
***Gpx3***	intrapulmonary airways	1.0 ± 0.1	0.9 ± 0.1	0.8 ± 0.1	1.0 ± 0.1	1.0 ± 0.1	1.0 ± 0.1
	tracheal epithelial cells	1.0 ± 0.1	0.3 ± 0.1	0.6 ± 0.1	0.3 ± 0.1	0.3 ± 0.1	0.4 ± 0.1
***Gr***	intrapulmonary airways	1.0 ± 0.1	1.3 ± 0.1	1.1 ± 0.1	1.5 ± 0.1	1.0 ± 0.1	1.5 ± 0.1
	tracheal epithelial cells	1.0 ± 0.2	1.1 ± 0.4	1.5 ± 0.3	0.9 ± 0.3	0.8 ± 0.2	0.9 ± 0.4
***HO-1***	intrapulmonary airways	1.0 ± 0.1	1.3 ± 0.1	1.1 ± 0.1	1.1 ± 0.1	1.1 ± 0.1	1.2 ± 0.1
	tracheal epithelial cells	1.2 ± 0.3	1.0 ± 0.2	1.1 ± 0.2	1.1 ± 0.2	1.1 ± 0.4	1.1 ± 0.3
***Mcp-1***	intrapulmonary airways	1.0 ± 0.6	12 ± 5	11 ± 4	10 ± 4	8 ± 3	11 ± 4
	tracheal epithelial cells	1.1 ± 0.3	1.2 ± 0.3	1.8 ± 0.6	0.4 ± 0.1	0.5 ± 0.2	1.1 ± 0.5
***KC***	intrapulmonary airways	1.0 ± 0.3	2.1 ± 0.6	1.7 ± 0.3	1.9 ± 0.3	1.1 ± 0.2	1.5 ± 0.3
	tracheal epithelial cells	1.7 ± 0.2	bld	bld	bld	bld	bld
***IL-6***	intrapulmonary airways	1.0 ± 1.3	7.9 ± 1.2	6.8 ± 1.4	8.5 ± 1.0	4.3 ± 0.5 *	5.7 ± 0.8
	tracheal epithelial cells	1.2 ± 0.3	1.5 ± 0.6	2.4 ± 1.3	3.1 ± 1.7	0.6 ± 0.3	3.5 ± 1.8
***IL-13***	intrapulmonary airways	1.0 ± 0.4	52 ± 8	40 ± 7	48 ± 7	34 ± 4 *	48 ± 7
	tracheal epithelial cells	1.1 ± 0.3	41 ± 14	27 ± 7	20 ± 9	26 ± 10	31 ± 10
***IL-17a***	intrapulmonary airways	1.0 ± 2.9	13 ± 3	13 ± 3	11 ± 1	8 ± 2	19 ± 4
	tracheal epithelial cells	bld	bld	bld	bld	bld	bld
***Muc5ac***	intrapulmonary airways	1.0 ± 0.3	44 ± 11	29 ± 4	52 ± 9	26 ± 5	82 ± 25
	tracheal epithelial cells	1.1 ± 0.3	35 ± 10	101 ± 15 **	38 ± 11	39 ± 23	39 ± 20
***Muc5b***	intrapulmonary airways	1.0 ± 0.2	31 ± 5	18 ± 3 *	32 ± 4	20 ± 2	39 ± 11
	tracheal epithelial cells	1.1 ± 0.2	2.2 ± 0.6	2.2 ± 0.2	2.1 ± 0.2	2.2 ± 0.4	2.4 ± 0.4

Data are presented as mean ± SEM (*n* = 4–8). The OVA/CBNP groups were compared to the OVA/H_2_O group and analyzed with the Mann Whitney U test. Significant changes were marked with * for *p* < 0.05 and ** for *p* < 0.01. bld = below limit of detection

## Supplementary Materials

The following are available online at http://www.mdpi.com/2079-4991/8/4/213/s1: Figure S1: Particle characteristics of P90-BaP and AS-PAH used in the present study. Figure S2: Carbon Black nanoparticle (CBNP)-exposure did not increase the lung wet weight in ovalbumin (OVA)-treated mice. Figure S3: CBNP did not increase the number of necrotic cells in the tracheal epithelium of OVA-treated mice. Figure S4: ATP did not increase the particle transport speed on the tracheal epithelium of OVA- and CBNP-treated mice. Table S1: The identified surface-bound polycyclic aromatic hydrocarbon (PAH) species of AS-PAH. Table S2: The primer and probe sequences used for quantitative polymerase chain reaction (PCR).
