# Supplementary material for: Low Dose Carbon Black Nanoparticle Exposure Does Not Aggravate Allergic Airway Inflammation in Mice Irrespective of the Presence of Surface Polycyclic Aromatic Hydrocarbons
Source: Nanomaterials (Basel). 2018 Mar 31;8(4):213. doi: 10.3390/nano8040213 (PMC5923543; doi:10.3390/nano8040213)
Supplement: Supplementary file 1 [file nanomaterials-08-00213-s001.pdf]

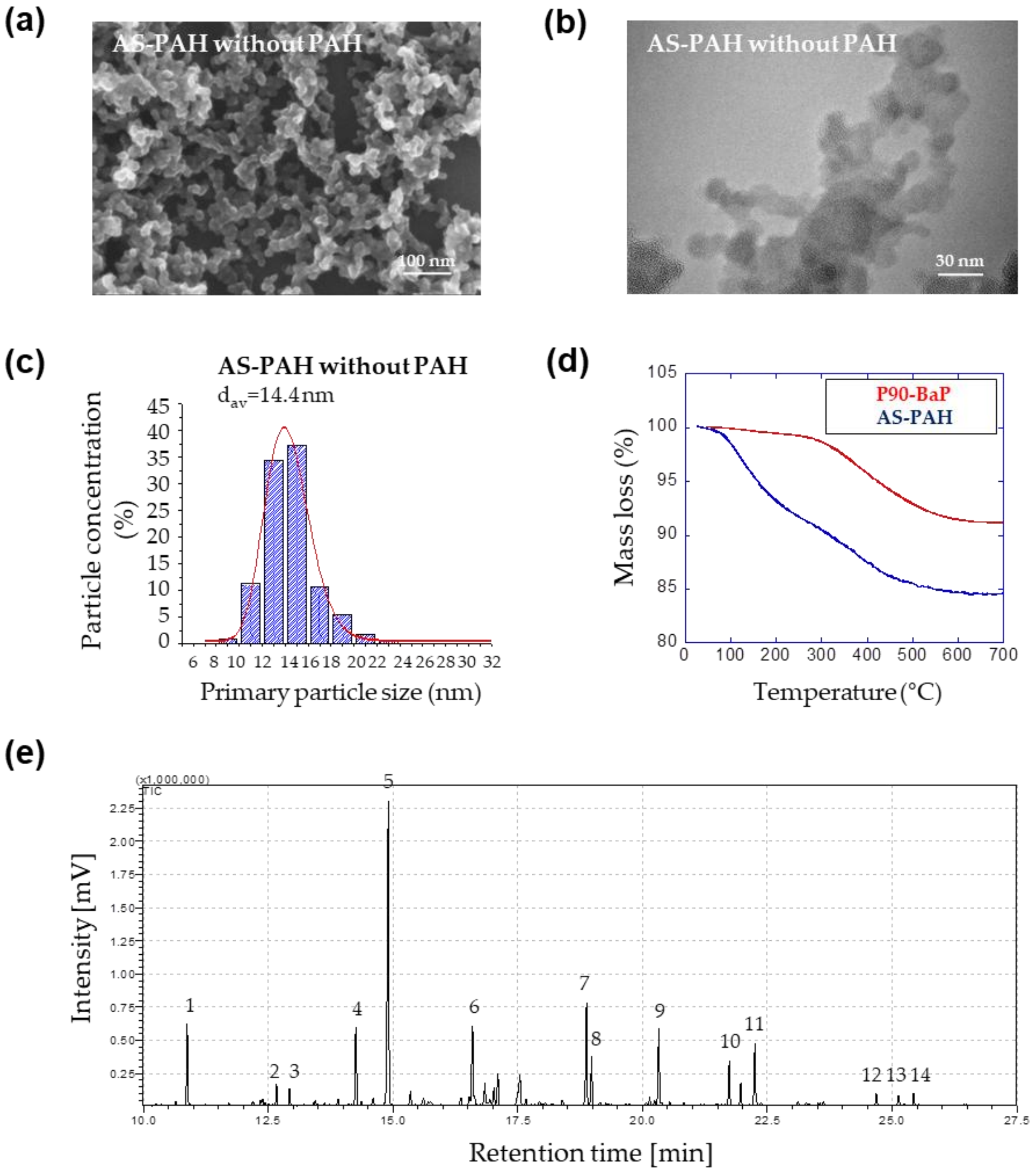

Figure S1: Particle characteristics of P90-BaP and AS-PAH used in the present study.

The images in (**a**) and (**b**) show the representative scanning electron microscopic images of PAH-depleted AS-PAH, (**a**) shows the scanning and (**b**) shows the transmission electron micrograph. The diagram in (**c**) displays the particle size distributions of AS-PAH without PAH. A representative thermogram of P90-BaP and AS-PAH is shown in diagram (**d**). The mass loss of P90-BaP is shown by red line and the mass loss of AS-PAH is shown by blue line. The image in (**e**) shows a representative GC/MS chromatogram of PAHs from an AS-PAH sample.

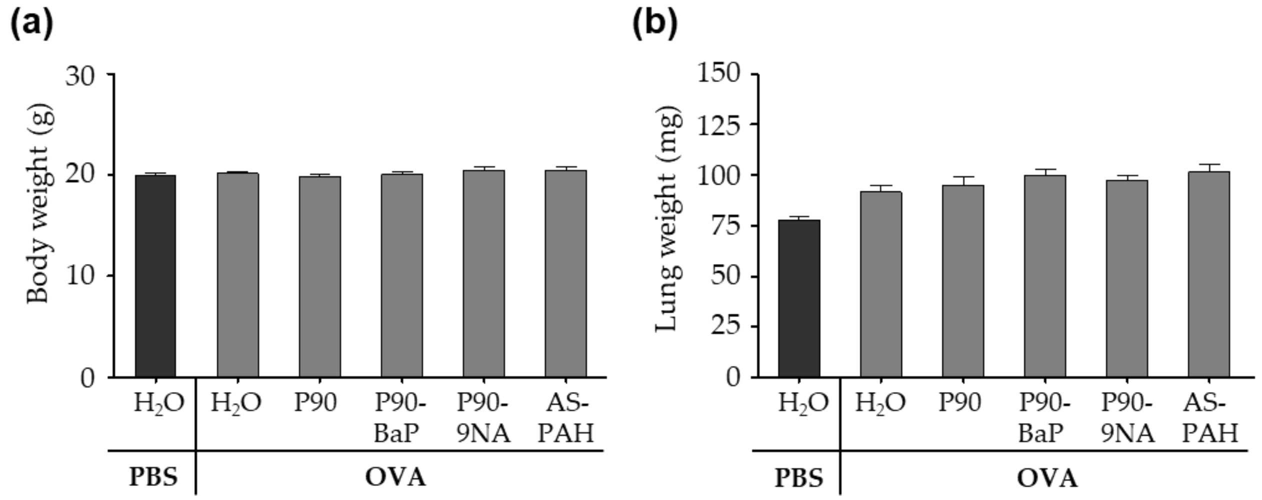

Figure S2: The CBNP exposure did not increase the wet weight of the lung in OVA-treated mice.

The diagrams show the final body weight (**a**) and the wet weight of the left lung (**b**) of mice. The results are presented as the mean ± SEM. (n = 13–18), $p < 0.05$ was considered statistically significant using the Mann Whitney U test.

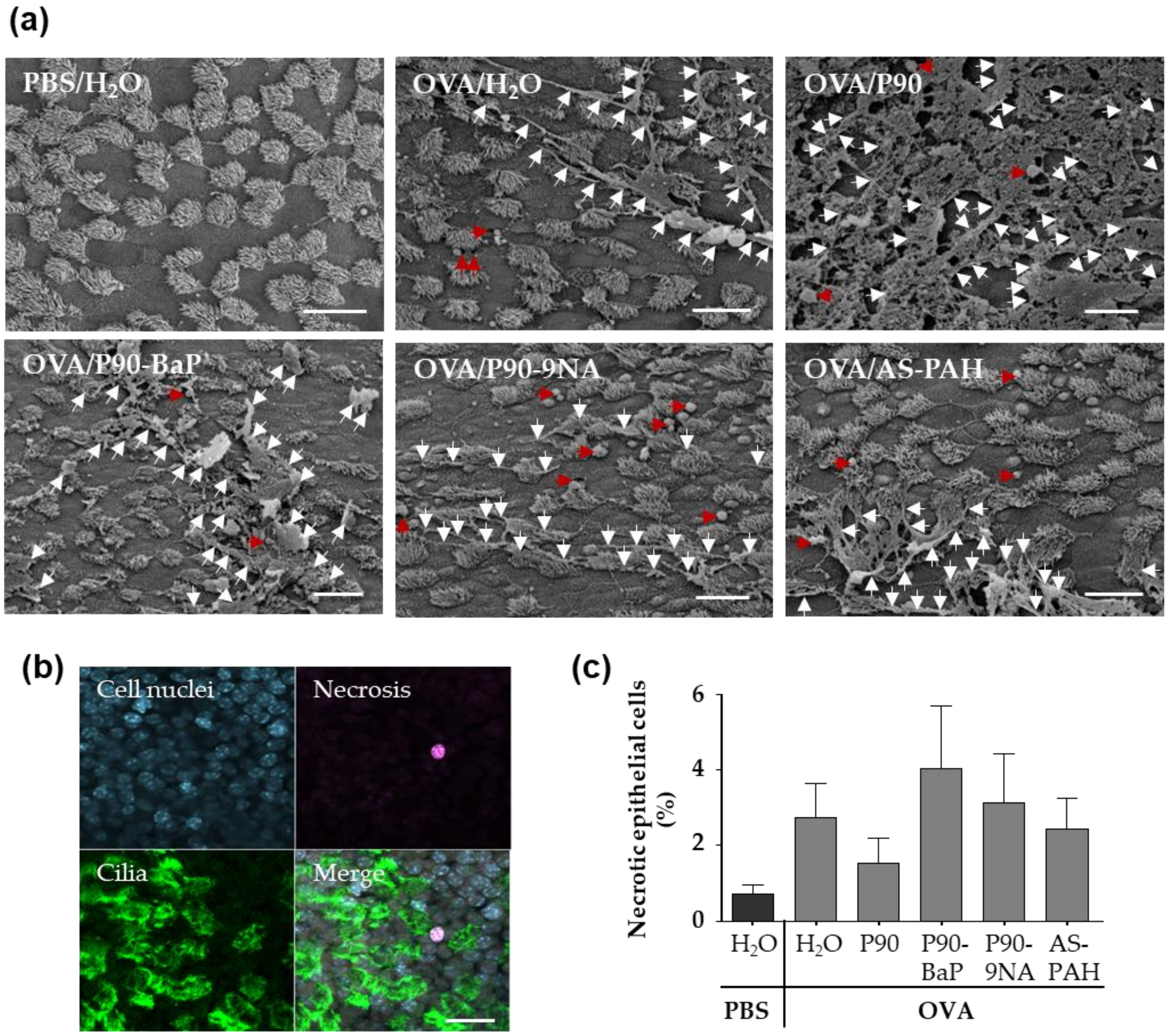

Figure S3: The CBNP did not increase the number of necrotic cells in the tracheal epithelium of OVA-treated mice.

The images in (**a**) show the tracheal epithelium after H_2O or CBNP exposure. The white arrows indicate mucus and the red arrows indicate dead cells on the the epithelium. (n = 3–4) The images in (**b**) show the stained tracheal epithelium, the cell nuclei are stained with Hoechst dye (blue), the necrotic cells are stained with Ethidium homodimer-1 (pink), and the cilia are labeled with a fluorescent-labeled anti-acetyled α-tubulin antibody. The quantification of necrotic cells is presented in (**c**) as a percentage of the total cell numbers. Mean ± SEM. (n = 4–6).

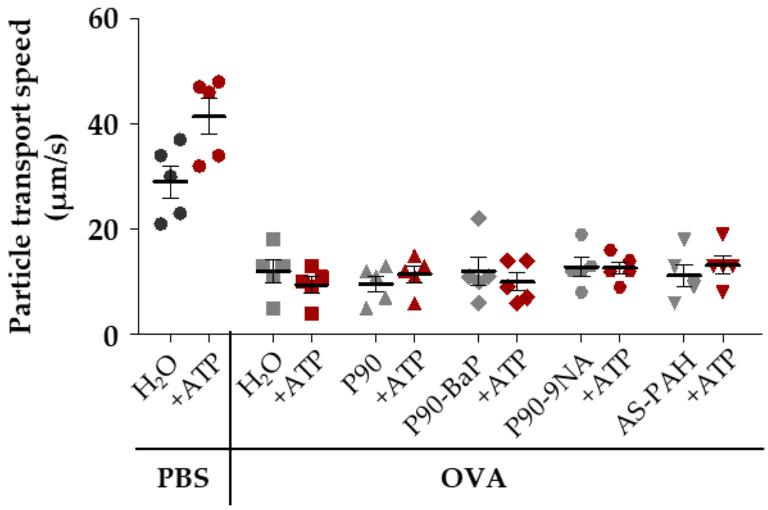

Figure S4: The ATP did not increase the particle transport speed on the tracheal epithelium of OVA- and CBNP-treated mice.

The diagram shows the mean particle transport speed after exposure to H_2O or CBNP (grey) and during the additional exposure to ATP (red). Each data point represents a single experiment. The lines with bars represent the mean ± SEM.

Table S1: The identified surface-bound PAH species of AS-PAH.

Number	Compound	Retention Time (min)	Molecular Weight (g/mol)	Rings	Reference
1	Naphthalene	10.875	128	2	NIST
2	1-methyl-naphthalene	12.683	146	2	NIST
3	2-methyl-naphthalene	12.942	146	2	NIST
4	Biphenylene	14.275	152	3	NIST
5	Acenaphthylene	14.908	152	3	R
6	Fluorene	16.608	166	3	R
7	Phenanthrene	18.883	178	3	NIST
8	Anthracene	18.992	178	3	R
9	4H-cyclopenta[def]phenanthrene	20.333	190	3	NIST
10	Fluoranthene	21.742	202	3	R
11	Pyrene	22.258	202	4	R
12–14	Cyclopenta[cd]pyrene/ Benzo[ghi]fluoranthene	24.632–25.435	226	5	NIST

The PAH were analyzed with GC/MS and identified by reference substances (R) or by the National Institute of Standards and Technology Database (NIST). The PAH compounds with numbers 12–14 are represented as isomers.

Table S2: The primer and probe sequences used for the quantitative PCR.

Target	NM Number	Sequence Primer Sense (5´-3´)	Sequence Primer Antisense (5´-3´)	Sequence TaqMan FAM-TAMRA (5´-3´)	Used For
Cyp1a1	009992.4	TTCCTGTCCTCCGTTACCTG	CCTGTCCTGACAATGCTCAA	CTTCCCTGGATGCCTTCAAGGACTTGAATG	both*
Cyp1b1	009994.1	TTACGGACATCTTCGGAGCC	AACCTGGTCCAACTCAGCC	CCTTTCCACCGCGCTGCTGTGGCTG	tec
		AGGATGTGCCTGCCACTATT	AGCTGGAGAATCGCATTGAT		ia
Gpx3	008161.3	CAGGCGAGAACTCGGAGATA	AGCGGATGTCATGGATCTTC	CAAGTATGTTCGACCAGGTGGGGGCTTTG	both*
Gr	010344.4	TCGGAATTCATGCACGATCAC	TGTTCAGGCGGCTCACATAG	GGCTTTCAAAGCTGTGAGGGTAAATTCAGTTGG	both*
HO-1	010442.2	AGAAGAGGCTAAGACCGCCT	CTCTGACGAAGTGACGCCAT	GTGTTCCTCTGTCAGCATCACCTGCAGCTCC	both*
IL-6	031168.1	CTCCCAACAGACCTGTCTATAC	GTGCATCATCGTTGTTCATAC	TGCCATTGCACAACTCTTTTCTCATTTCCACG	both*
IL-13	008355.3	GGAGCTTATTGAGGAGCTGAG	CAGGGAATCCAGGGCTACAC	CATCACACAAGACCAGACTCCCCTGTGC	tec
		Quantitect-Qiagen QT00099554			ia
IL-17A	010552.3	TCAGACTACCTCAACCGTTCC	CTTTCCCTCCGCATTGACAC	CACCCTGGACTCTCCACCGCAATGAAG	tec
		TGTGAAGGTCAACCTCAAAGTCT	GAGGGATATCTATCAGGGTCTTCAT		ia
KC (Cxcl1)	008176.3	CAGACCATGGCTGGGATTC	GAACCAAGGGAGCTTCAG	CCTCGCGACCATTCTTGAGTGTGGCTATGAC	both*
Mcp-1	011333.3	TAGGCTGGAGAGCTACAAGAG	TCTGGACCCATTCCTTCTTG	GCAGCAGGTGTCCAAAGAAGCTGTAGTTTTTG	tec
		GGCTCAGCCAGATGCAGTTA	GCTGCTGGTGATCCTCTTGT		ia
Muc5ac	010844.1	AGATCTCCGTGGTCTGGAA	GGTACAACTTTCCATTGACG	CCCATCTTTGATGAGGGCACAATGCAGTG	tec
		Quantitect-Qiagen QT01744575			ia
Muc5b	028801.2	CAGAAACTGGAGCTGG	GGATATGGAGTCACTATACA	CCTCCGTGGCCTTGCTCATGGTGTG	tec
		GTGCCTTGTCTCAGTCCCTC	TCTGACTGTCTCCGGTGAGT		ia
Rpl32	172086.2	AAAATTAAGCGAAACTGGCG	ATTGTGGACCAGGAACTTGC	CATTGACAACAGGGTGCGGAGAAGGTTC	both*

* Intrapulmonary airways and tracheal epithelial cells, tec = tracheal epithelial cells, ia = intrapulmonary airways.
